# Supplementary material for: BHLHE41/DEC2 Expression Induces Autophagic Cell Death in Lung Cancer Cells and Is Associated with Favorable Prognosis for Patients with Lung Adenocarcinoma
Source: Int J Mol Sci. 2021 Oct 26;22(21):11509. doi: 10.3390/ijms222111509 (PMC8584041; doi:10.3390/ijms222111509)
Supplement: Supplementary file 1 [file ijms-22-11509-s001.zip › ijms-1298375-supplementary/ijms-1298375_Supp figs.pdf]

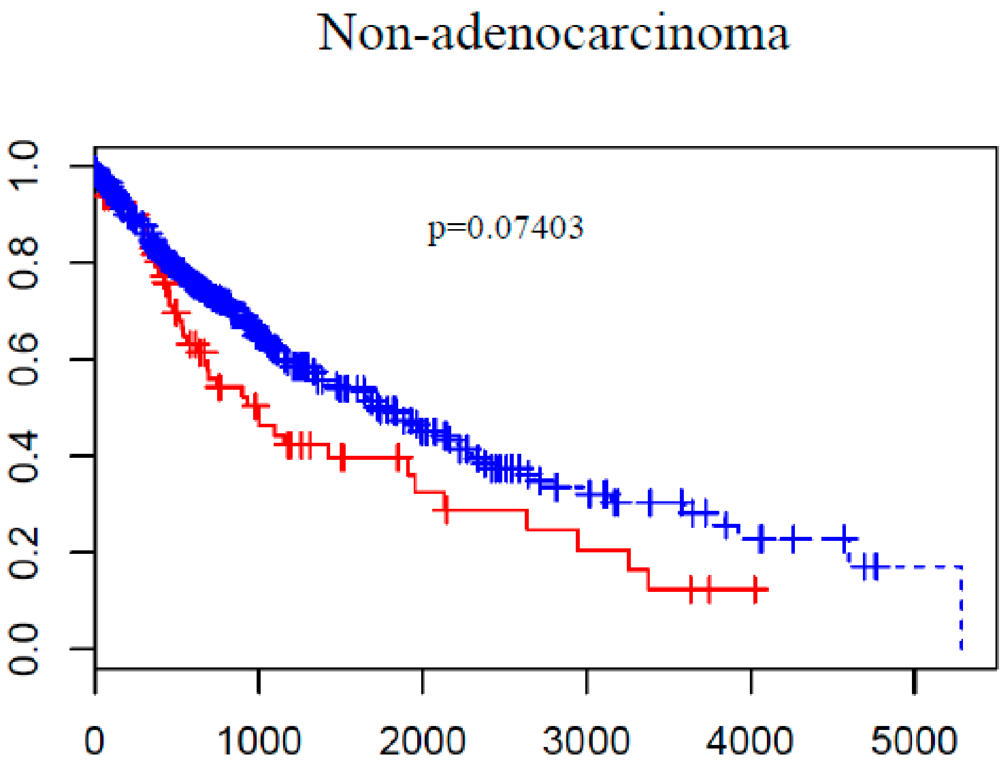

Figure S1: In silico analysis of BHLHE41 expression and Kaplan–Meier plot data of LUSC)

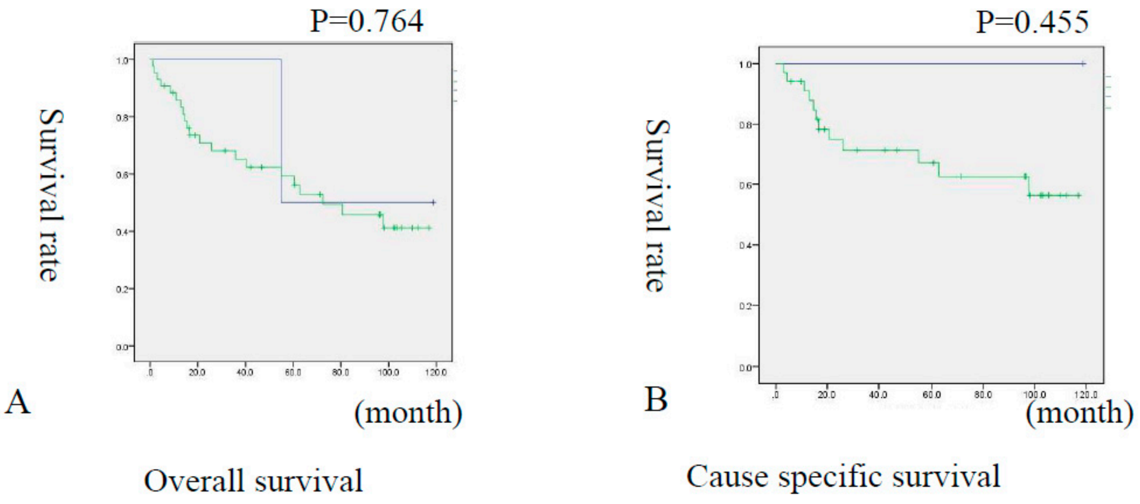

Figure S2: Overall survival and cause-specific survival of the patients according to their expression of BHLHE41 in the Non-LUAD group

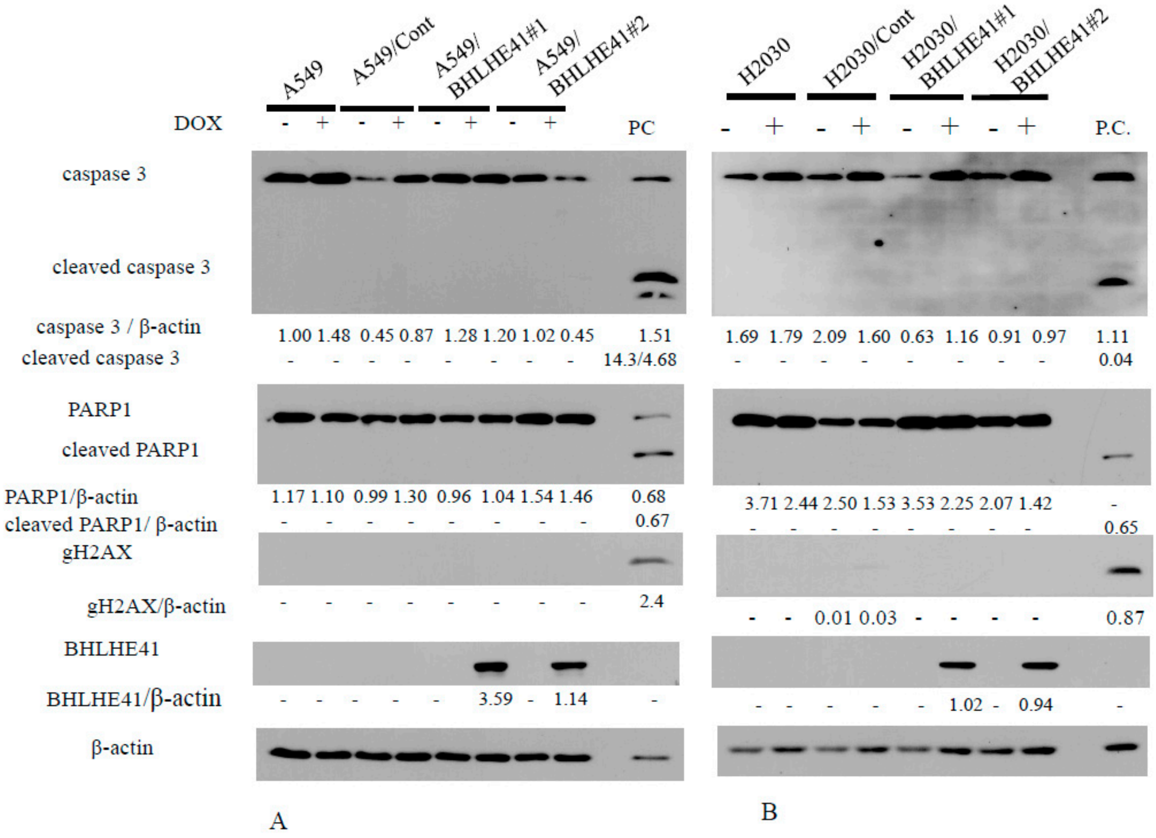
A549
A549/Cont
A549/ BHLHE41#1
A549/ BHLHE41#2
DOX
PC
caspase 3
cleaved caspase 3
caspase 3 / β-actin
cleaved caspase 3
1.00 1.48 0.45 0.87 1.28 1.20 1.02 0.45 1.51
14.3/4.68
PARP1
cleaved PARP1
PARP1/β-actin
cleaved PARP1/ β-actin
1.17 1.10 0.99 1.30 0.96 1.04 1.54 1.46 0.68
0.67
gH2AX
gH2AX/β-actin
2.4
BHLHE41
BHLHE41/β-actin
3.59
1.14
β-actin
H2030
H2030/Cont
H2030/ BHLHE41#1
H2030/ BHLHE41#2
P.C.
1.69 1.79 2.09 1.60 0.63 1.16 0.91 0.97 1.11
0.04
3.71 2.44 2.50 1.53 3.53 2.25 2.07 1.42
0.65
0.01 0.03
0.87
1.02
0.94
A
B

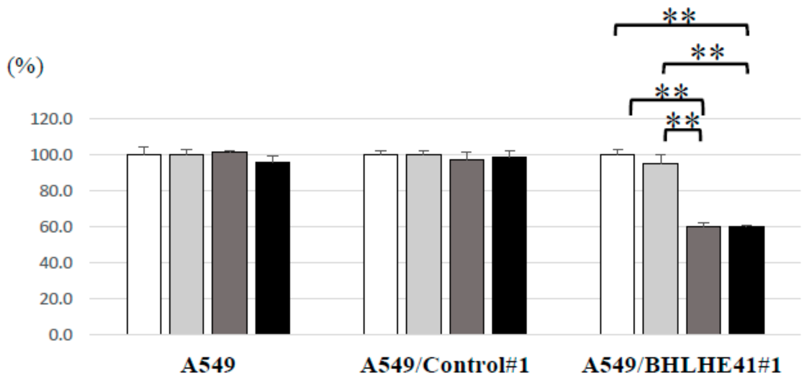

ZVAD	-	+	-	+		-	+	-	+		-	+	-	+
DOX	-	-	+	+		-	-	+	+		-	-	+	+

C

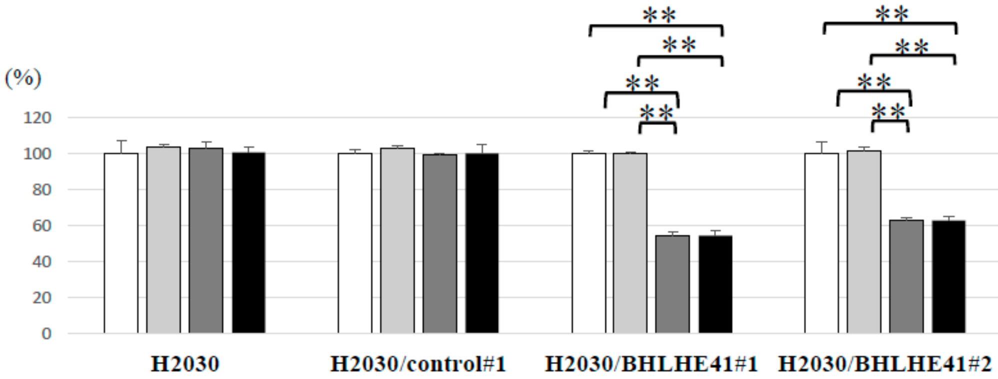

ZVAD	-	+	-	+		-	+	-	+		-	+	-	+		-	+	-	+
DOX	-	-	+	+		-	-	+	+		-	-	+	+		-	-	+	+

D

Figure S3: Immunoblotting of apoptosis-related proteins in the absence or presence of DOX and cell survival assay in the presence of Z-VAD-FMK

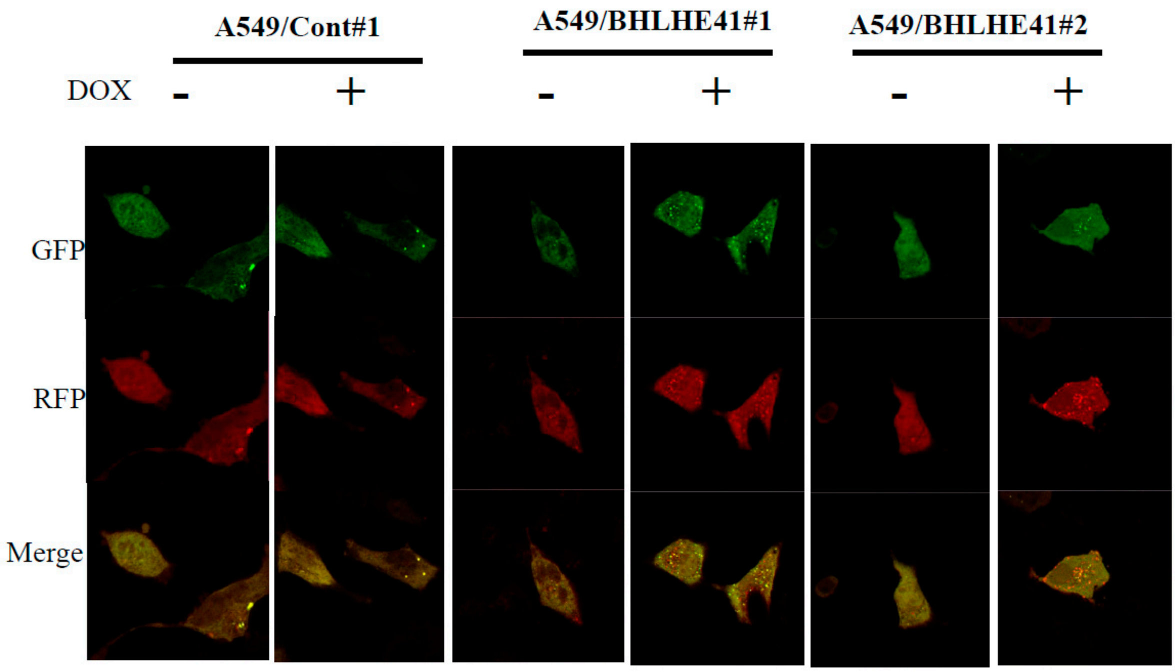

Figure S4 : ftLC3 readout in the presence and absence BHLHE41 expression

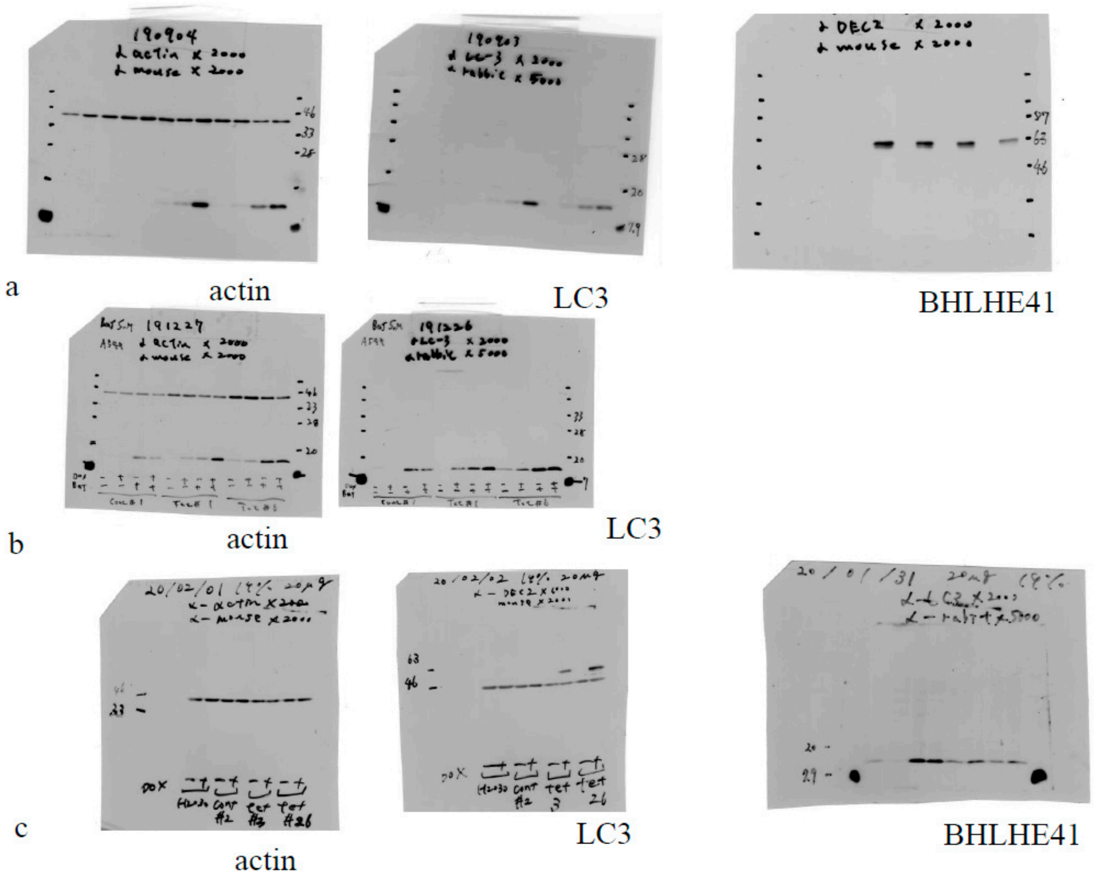

Figure S5: whole blot film of Figure 5

(A)

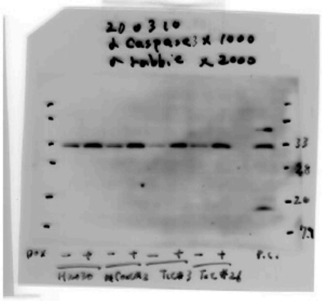

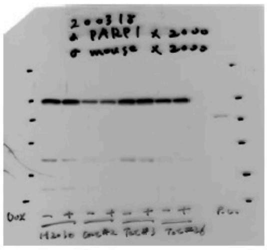

PARP1

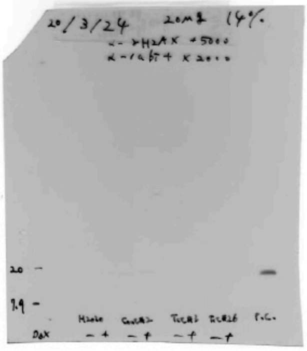

gH2AX

caspase3

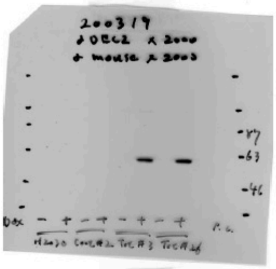

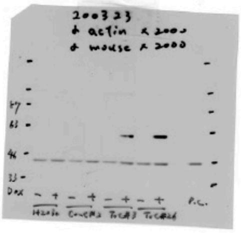

actin

BHLHE41

(B)

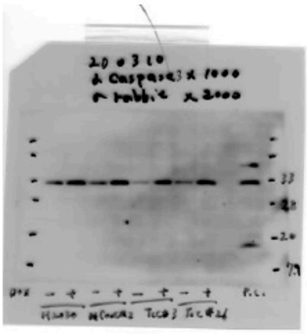

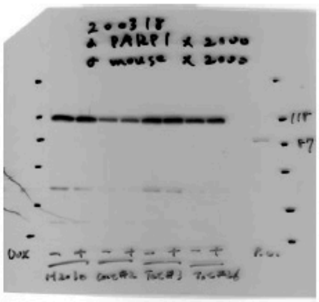

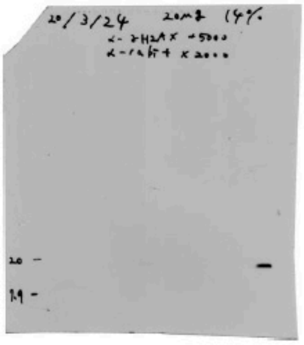

PARP1

caspase3

gH2AX

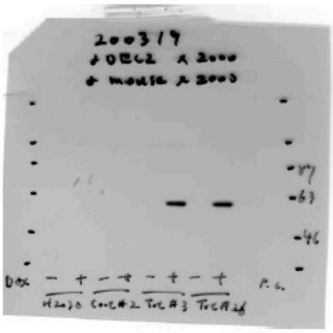

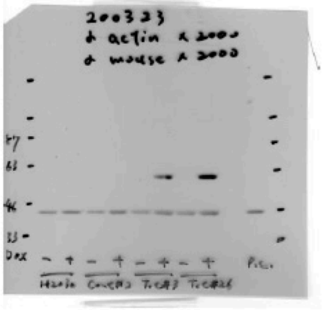

BHLHE41

actin

Figure S6: whole blot film of Figure S3a and S3b
