# Supplementary material for: BHLHE41/DEC2 Expression Induces Autophagic Cell Death in Lung Cancer Cells and Is Associated with Favorable Prognosis for Patients with Lung Adenocarcinoma
Source: Int J Mol Sci. 2021 Oct 26;22(21):11509. doi: 10.3390/ijms222111509 (PMC8584041; doi:10.3390/ijms222111509)
Supplement: Supplementary file 1 [file ijms-22-11509-s001.zip › ijms-1298375-supplementary/ijms-1298375_Supp tables.pdf]

Clinicopathologic Factors		Expression of BHLHE41					
		Positive		Negative		P value	
		N=17	(%)	N=43	(%)		
Total		17	(9.6)	160	(90.4)		
Age	<70years	9	(11.1)	72	(88.9)	0.532	
	≥70years	8	(9.9)	88	(49.7)		
Gender	female	10	(12.3)	71	(40.1)	0.256	
	male	7	(8.6)	89	(50.3)		
Tumor size	≤30mm	14	(17.3)	95	(53.7)	0.064	
	>30mm	3	(3.7)	65	(36.7)		
Pleural invasion	No	16	(19.8)	117	(66.1)	0.044	*
	Yes	1	(1.2)	43	(24.3)		
Pulmonary metastasis	No	17	(21.0)	150	(84.7)	0.354	
	Yes	0	(0.0)	10	(5.6)		
T factor	T1	15	(18.5)	85	(48.0)	0.005	*
	≥T2	2	(2.5)	75	(42.4)		
N factor	No	15	(18.5)	121	(68.4)	0.196	
	Yes	2	(2.5)	39	(22.0)		
Stage	IA	14	(17.3)	69	(39.0)	0.002	*
	≥IB	3	(3.7)	91	(51.4)		
Histology	Adenocarcinoma	15	(18.5)	117	(66.1)	0.141	
	Others	2	(2.5)	43	(24.3)		

Table S1 : Correlation between BHLHE41 expression in NSCLC and clinicopathologic findings. Histological classification AIS (adenocarcinoma in situ) was followed to TNM classification 8th edition. * indicates significant correlation (P<0.05)

Clinicopathologic Factors		Expression of BHLHE41				
		Positive		Negative		P value
		N=2	%	N=43	%	
Total		2	(4.4)	43	(95.6)	
Age	<70years	1	(4.5)	21	(95.5)	0.744
	≥70years	1	(4.3)	22	(95.7)	
Gender	female	0	(0.0)	4	(100.0)	0.828
	male	2	(4.9)	39	(95.1)	
Tumor size	≤30mm	1	(5.0)	19	(95.0)	0.697
	>30mm	1	(4.0)	24	(96.0)	
Pleural invasion	No	1	(3.6)	27	(96.4)	0.618
	Yes	1	(5.9)	16	(94.1)	
Pulmonary metastasis	No	2	(4.8)	40	(95.2)	0.87
	Yes	0	(0.0)	3	(100.0)	
T factor	T1	1	(6.7)	14	(93.3)	0.561
	≥T2	1	(3.3)	29	(96.7)	
N factor	No	2	(7.1)	26	(92.9)	0.382
	Yes	0	(0.0)	17	(100.0)	
Stage	IA	1	(12.5)	7	(87.5)	0.327
	≥IB	1	(2.7)	36	(97.3)	

TableS2 : Correlation between clinicopathologic factors and BHLHE41 expression in non-adenocarcinoma

		n	dead	alive	Univariate HR (95%CI)	Univariate *P* value	Multivariate HR (95%CI)	Multivariate *P* value
Age	≥70 years	23	12	11	1.47	0.37	1.73	0.22
	<70	22	10	12	(0.63 - 3.44)		(0.72 - 4.14)	
Gender	male	41	20	21	0.97	0.97	0.87	0.85
	female	4	2	2	(0.23 - 4.16)		(0.20 - 3.79)	
Tumor size	≥30mm	25	11	14	0.72	0.45		
	<30mm	20	11	9	(0.31 - 1.67)			
Pleural invasion	Yes	17	13	4	2.89	0.015*		
	No	28	9	19	(1.23 - 6.77)			
Plulmonary metastasis	Yes	3	1	2	0.64	0.67		
	No	42	21	21	(0.086 - 4.84)			
T factor	≥T2	30	16	14	1.71	0.27		
	T1	15	6	9	(0.67 - 4.37)			
N factor	N1/2	17	9	8	1.65	0.25		
	N0	28	13	15	(0.70 - 3.86)			
Pathological stage	≥IB	37	19	18	1.95	0.29	2.33	0.21
	IA	8	3	5	(0.57 - 6.59)		(0.63 - 8.66)	
BHLHE41	negative	43	21	22	1.36	0.77	0.98	0.99
	positive	2	1	1	(0.18 - 10.13)		(0.12 - 8.08)	

Table S3 : Univariate and multivariate COX regression analysis in patients with non-adenocarcinoma for overall survival. In multivatiate analysis same factors as Table.3. * indicates significant correlation (P<0.05)
